# A hypothesis on the role of perturbation size on the human sensorimotor adaptation

**DOI:** 10.3389/fncom.2014.00028

**Published:** 2014-03-11

**Authors:** Fatemeh Yavari, Farzad Towhidkhah, Mohammad Darainy

**Affiliations:** ^1^Biomedical Engineering Department, Amirkabir University of TechnologyTehran, Iran; ^2^Department of Psychology, McGill UniversityMontreal, QC, Canada

**Keywords:** adaptation, perturbation amplitude, error size, sensory recalibration, internal model

## Introduction

Some evidence suggests that depending on the size of error produced by a perturbation, distinct learning mechanisms and neural structures are employed in the brain (Kluzik et al., 2008; Criscimagna-Hemminger et al., 2010; Gibo et al., 2013). Here, based on some existing evidence, we propose a hypothesis about the potential adaptation mechanisms which may be employed in the brain based on the perturbation magnitude. In the following sections, we first briefly explain the proposed hypothesis. Then a short description about the resolution of hand proprioceptive sensory is presented. In this hypothesis, the size of error is assessed relative to the resolution of proprioceptive sensory. Next, the empirical evidence supporting the proposed hypothesis are shortly described.

## The hypothesis

**Figure 1:**
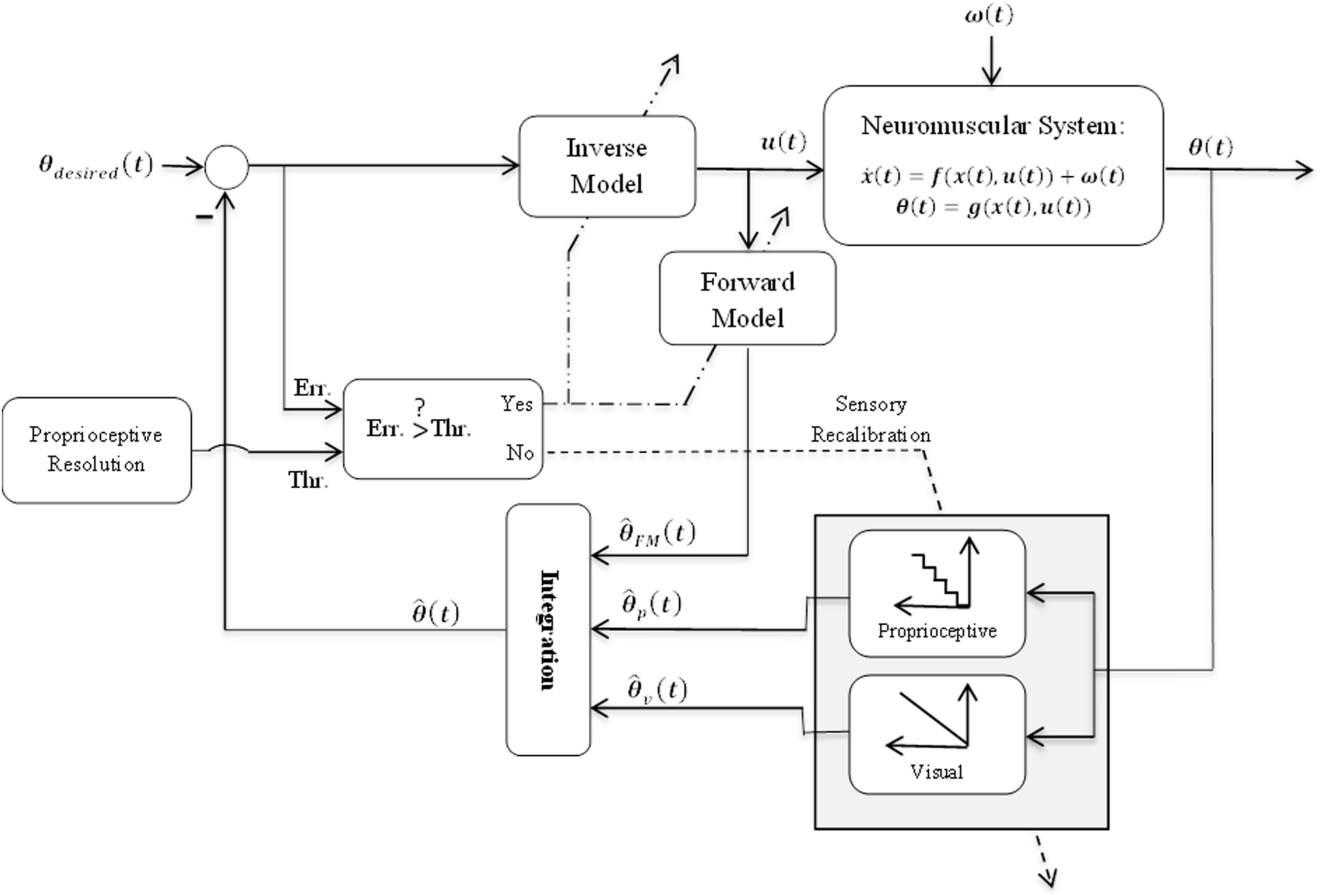
**Schematic representation of the proposed hypothesis**. The general structure of this model has been borrowed from other studies e.g., (Shadmehr and Krakauer, 2008). θ(*t*), $\hat{\theta }$_*FM*_(*t*), $\hat{\theta }$_*p*_(*t*), and $\hat{\theta }$_*v*_(*t*) are respectively system output and its estimations by forward model, proprioceptive sensory, and visual sensory. $\hat{\theta }$(*t*) is final estimation of system output obtained from integration. Dashed and dot-dashed lines show sensory recalibration and internal models' (IMs') adaptation, respectively.

Our hypothesis schematically represented in Figure 1 is as follows:

1- For small perturbation amplitude compared to proprioceptive sensory resolution, the produced movement error (Err. in Figure 1) will be small as well. Small error does not often result in subject's awareness (Cressman and Henriques, 2009; Criscimagna-Hemminger et al., 2010). In this condition, the brain may consider the perturbation resulting from an internal source and compensate it with recalibration of proprioceptive sensory. This may be expressed by shifting the input-output relationship of proprioceptive sensory module (i.e., Proprioceptive block in Figure 1). The input-output relationship of this module has been modeled with a quantization (staircase) function to represent the limited resolution.
2- For large perturbation amplitude, the produced movement error will be large as well, which typically make subject aware of the perturbation (Malfait and Ostry, 2004). In this case the assumption is that the perturbation may be caused by an external source and the brain may need to form/update internal forward and/or inverse models of the new dynamics to reduce movement errors.

## Resolution of proprioceptive sensory

It is possible to infer about the resolution of proprioceptive sensory based on some of previous studies. Diedrichsen et al. (2010) moved the subject's hand passively using a robotic arm along a trajectory deviated 8° to the left or right of the subjects' body midline. In the absence of visual feedback, subjects were not able to guess the direction of this deviation. In another study (Farrer et al., 2003), the experimenter moved subject's hand by pulling a rod connected to a joystick. Subjects had no direct view of their hand; instead a virtual hand image provided the visual feedback for them. The visual feedback was deviated either to the right or left relative to the actual hand movement by a certain angular value (0, 5, 10, 15, 20, 30, 40, or 50°) in each trial. At the end of each movement, subjects had to indicate if their movement and the visual feedback were at the same place. They were not able to detect the deviation when it was less than 5° (Figure 2. in Farrer et al., 2003). Also, Darainy et al. (2013) observed that during passive hand movements perceptual boundary was at the left of the midline. Based on the observations in the above mentioned studies and some others (Cressman and Henriques, 2009; Fuentes et al., 2011), it can be suggested that resolution of proprioceptive sensory is about 5° (in the midline direction). On the other hand, there are some evidence supporting this notion that proprioceptive sensory is more precise in front-back direction than left-right (van Beers et al., 2002; Wilson et al., 2010). Therefore it seems plausible to infer that maximum resolution of proprioceptive sensory is in the midline direction.

## Evidence supporting the proposed hypothesis

Some of the observations which can be explained based on this hypothesis are given in the following:

- Based on the proposed hypothesis, adaptation to an abrupt perturbation, which produces large errors, results in formation of an IM in the brain, while adaptation to a gradual perturbation is probably not dependent on IMs. Cerebellum is one of the main candidate brain regions to contain IMs, specifically internal forward models (see Yavari et al., 2013 for a review). It has already been demonstrated that individuals with cerebellar damage have difficulties in adapting to an abrupt force field during hand reaching movements (Smith and Shadmehr, 2005); however when that perturbation was imposed gradually they are usually able to adapt their movements (Criscimagna-Hemminger et al., 2010; Izawa et al., 2012). These observations confirm dependency of adaptation in presence of large, but not small errors on cerebellum and are in line with the proposed hypothesis.
- It has been observed that sudden and gradual introduction of perturbations—which result in large and small errors, respectively—produce different generalization patterns. Motor memories produced by abrupt perturbations are in an extrinsic coordinate system and generalize to the untrained arm (Criscimagna-Hemminger et al., 2003; Malfait and Ostry, 2004), whereas gradual presentation of perturbations cause adaptation in intrinsic arm coordinates that does not transfer to the other arm (Malfait and Ostry, 2004; Wilson et al., 2010). Also it has been observed that gradual perturbations lead to more robust generalization when using the trained arm in a different context, while this generalization is smaller in response to a sudden perturbation (Kluzik et al., 2008). These observations can be explained based on the proposed hypothesis as follows: the brain forms an IM of the perturbation in response to large errors (in an extrinsic coordinate system). The created model would be applicable in performing movements with another hand in the presence of the same perturbation. On the other hand, gradual presentation of the perturbation results in sensory recalibration which is specific to the trained arm (intrinsic arm coordinates). This explains the generalization pattern produced by small errors.
- Subjects showed almost the same size of aftereffect when adapted to gradual and abrupt perturbations; however washout rate was significantly higher in the abrupt group (Kluzik et al., 2008). On the other hand, functional imaging and computational studies support the existence of multiple IMs in the brain which are activated based on the context (Haruno et al., 2001; Imamizu et al., 2003, 2004). Having this point in mind, the mentioned observation may be explained as follows: adaptation to an abrupt perturbation results in formation of an IM in the brain. Eliminating the perturbation causes aftereffects which will not last for long because the brain rapidly switches back to the suitable IM for the condition with no perturbation. This may not be the case for small errors.
- Sensory recalibration due to adaptation to small errors has been observed in some previous studies (Cressman and Henriques, 2009).

## Summary

We presented a hypothesis about the possible adaptation mechanisms employed in the brain based on error size. The proposed hypothesis can help to provide a better understanding of motor adaptation mechanism in brain. Further validation of the hypothesis requires more investigations and experiments. For example, adaptation in response to a gradual perturbation can be compared in deafferented subjects, cerebellar patients, and healthy individuals. This comparison may be performed regarding generalization patterns to untrained hand or to other contexts with the same hand, adaptation rate, wash-out rate, etc. It has been shown that deafferented individuals were able to adapt their reaches to altered visual feedback of the hand (Ingram et al., 2000; Bernier et al., 2006; Miall and Cole, 2007). Adaptation in these subjects may show different features compared to healthy ones.

## References

[B1] Bernier P.-M. Chua R. Bard C. Franks I. M. (2006). Updating of an internal model without proprioception: a deafferentation study. Neuroreport 17, 1421–1425 10.1097/01.wnr.0000233096.13032.34 16932151

[B2] Cressman E. K. Henriques D. Y. (2009). Sensory recalibration of hand position following visuomotor adaptation. J. Neurophysiol. 102, 3505–3518 10.1152/jn.00514.2009 19828727

[B3] Criscimagna-Hemminger S. E. Bastian A. J. Shadmehr R. (2010). Size of error affects cerebellar contributions to motor learning. J. Neurophysiol. 103, 2275–2284 10.1152/jn.00822.2009 20164398 PMC2853280

[B4] Criscimagna-Hemminger S. E. Donchin O. Gazzaniga M. S. Shadmehr R. (2003). Learned dynamics of reaching movements generalize from dominant to nondominant arm. J. Neurophysiol. 89, 168–176 10.1152/jn.00622.2002 12522169

[B5] Darainy M. Vahdat S. Ostry D. J. (2013). Perceptual learning in sensorimotor adaptation. J. Neurophysiol. 110, 2152–2162 10.1152/jn.00439.2013 23966671 PMC4073967

[B6] Diedrichsen J. White O. Newman D. Lally N. (2010). Use-dependent and error-based learning of motor behaviors. J. Neurosci. 30, 5159–5166 10.1523/JNEUROSCI.5406-09.2010 20392938 PMC6632748

[B7] Farrer C. Franck N. Paillard J. Jeannerod M. (2003). The role of proprioception in action recognition. Conscious. Cogn. 12, 609–619 10.1016/S1053-8100(03)00047-3 14656504

[B8] Fuentes C. T. Mostofsky S. H. Bastian A. J. (2011). No proprioceptive deficits in autism despite movement-related sensory and execution impairments. J. Autism Dev. Disord. 41, 1352–1361 10.1007/s10803-010-1161-1 21165765 PMC3118271

[B9] Gibo T. L. Criscimagna-Hemminger S. E. Okamura A. M. Bastian A. J. (2013). Cerebellar motor learning: are environment dynamics more important than error size? J. Neurophysiol. 110, 322–333 10.1152/jn.00745.2012 23596337 PMC3727069

[B10] Haruno M. Wolpert D. M. Kawato M. (2001). Mosaic model for sensorimotor learning and control. Neural Comput. 13, 2201–2220 10.1162/089976601750541778 11570996

[B11] Imamizu H. Kuroda T. Miyauchi S. Yoshioka T. Kawato M. (2003). Modular organization of internal models of tools in the human cerebellum. Proc. Natl. Acad. Sci. U.S.A. 100, 5461–5466 10.1073/pnas.0835746100 12704240 PMC154367

[B12] Imamizu H. Kuroda T. Yoshioka T. Kawato M. (2004). Functional magnetic resonance imaging examination of two modular architectures for switching multiple internal models. J. Neurosci. 24, 1173–1181 10.1523/JNEUROSCI.4011-03.2004 14762135 PMC6793589

[B13] Ingram H. A. van Donkelaar P. Cole J. Vercher J. L. Gauthier G. M. Miall R. C. (2000). The role of proprioception and attention in a visuomotor adaptation task. Exp. Brain Res. 132, 114–126 10.1007/s002219900322 10836641

[B14] Izawa J. Criscimagna-Hemminger S. E. Shadmehr R. (2012). Cerebellar contributions to reach adaptation and learning sensory consequences of action. J. Neurosci. 32, 4230–4239 10.1523/JNEUROSCI.6353-11.2012 22442085 PMC3326584

[B15] Kluzik J. Diedrichsen J. Shadmehr R. Bastian A. J. (2008). Reach adaptation: what determines whether we learn an internal model of the tool or adapt the model of our arm? J Neurophysiol 100, 1455–1464 10.1152/jn.90334.2008 18596187 PMC2544452

[B16] Malfait N. Ostry D. J. (2004). Is interlimb transfer of force-field adaptation a cognitive response to the sudden introduction of load? J. Neurosci. 24, 8084–8089 10.1523/JNEUROSCI.1742-04.2004 15371509 PMC6729794

[B17] Miall R. C. Cole J. (2007). Evidence for stronger visuo-motor than visuo-proprioceptive conflict during mirror drawing performed by a deafferented subject and control subjects. Exp. Brain Res. 176, 432–439 10.1007/s00221-006-0626-0 16874511

[B18] Shadmehr R. Krakauer J. W. (2008). A computational neuroanatomy for motor control. Exp. Brain Res. 185, 359–381 10.1007/s00221-008-1280-5 18251019 PMC2553854

[B19] Smith M. A. Shadmehr R. (2005). Intact ability to learn internal models of arm dynamics in Huntington's disease but not cerebellar degeneration. J. Neurophysiol. 93, 2809–2821 10.1152/jn.00943.2004 15625094

[B20] van Beers R. J. Wolpert D. M. Haggard P. (2002). When feeling is more important than seeing in sensorimotor adaptation. Curr. Biol. 12, 834–837 10.1016/S0960-9822(02)00836-9 12015120

[B21] Wilson E. T. Wong J. Gribble P. L. (2010). Mapping proprioception across a 2D horizontal workspace. PLoS ONE 5:e11851 10.1371/journal.pone.0011851 20686612 PMC2912297

[B22] Yavari F. Towhidkhah F. Ahmadi-Pajouh M. A. (2013). Are fast/slow process in motor adaptation and forward/inverse internal model two sides of the same coin? Med. Hypotheses 81, 592–600 10.1016/j.mehy.2013.07.009 23899631

